# Draft Genome Sequence of the Dye-Decolorizing and Nanowire-Producing Bacterium *Shewanella xiamenensis* BC01

**DOI:** 10.1128/genomeA.00721-14

**Published:** 2014-08-07

**Authors:** Yuzhe Li, I-Son Ng, Xia Zhang, Nan Wang

**Affiliations:** a Department of Chemical and Biochemical Engineering, College of Chemistry and Chemical Engineering, Xiamen University, Xiamen, China; b Department of Energy, Environmental and Chemical Engineering, Washington University in St. Louis, St. Louis, Missouri, USA; c Department of Chemical Engineering, National Cheng Kung University, Tainan, Taiwan

## Abstract

*Shewanella xiamenensis* BC01 is an important biodecolorizing and nanowire-producing bacterium which was found in Xiamen, China. Here, we present the draft genome sequence consisting of 4,677,169 bp (GC content, 46.21%) and 3,999 coded proteins. This information boosts insight into and understanding of the genetic evolution of *Shewanella* species.

## GENOME ANNOUNCEMENT

*Shewanella xiamenensis* sp. nov. was classified as a novel species because of its distinct characteristics (1). Recently, a new strain, *S. xiamenesis* BC01, was isolated in Xiamen, China. This strain is active in biodecolorization of textile azo dyes and production of nanowires in cells, which respond to electron transfer, thus accelerating azo dye reduction (2). Biodecolorization ability using a diverse range of electron acceptors for anaerobic respiration has been explored in *Shewanella oneidensis* MR-1 and *Shewanella decolorationis* S12 (3, 4). On the other hand, *bla*_OXA-48_, an emerging carbapenemase-encoding gene, was discovered in a chromosome initially found in *Klebsiella pneumoniae* (5). It also exists in *S. xiamenensis*, in which the wide range and high spreading speed of *bla*_OXA-48_ were demonstrated (6). However, further research revealed that such *bla*_OXA-48_-like genes do not exist in *S. oneidensis* MR-1 and *S. decolorationis* S12. Hence, the analysis of genome sequencing for this new species is of great importance.

At Yourgene Bioscience (Taiwan), high-throughput DNA sequencing of BC01 was conducted on the Illumina HiSeq 2500 with a 151-bp paired-end platform and an average insert size of 280 bp. A total of 15,442,652 reads were achieved, resulting in 394-fold genomic coverage. Reads were filtered to remove adapter sequences and achieve a quality trim of at least 35 bp. A draft genome was generated by *de novo* assembly, which was performed using a high-volume read-accommodating algorithm by storing data in de Bruijn graphs (7). This genome is a single circular chromosome of 4,677,169 bp, with a mean GC content of 46.21%. A total of 111 contigs with an *N*_50_ value of 95,445 and a largest contig size of 305,559 bp were constructed. Gene prediction using GeneMark (8) revealed a total of 3,999 open reading frames (ORFs). The predicted proteins were annotated by implementing a BLAST (blastp) search against the NCBI-NR database.

Whole-genome sequences were obtained from NCBI taxonomy for 37 strains from 26 type species of *Shewanella* and facilitated bioinformatics-based genome functional predictions and analyses. Through this process, we found that BC01 was closest to *S. decolorationis* S12 (77.2% of coding sequences with over 85% similarity) and *Shewanella* sp. MR-1 (73.6% of coding sequences with over 85% similarity), followed by *S. oneidensis* MR-4 (72.9% of coding sequences with over 85% similarity), findings which suggested the uniqueness of this strain. Further comparison revealed that BC01 and MR-1 are similar, with 3,423 known protein-encoding genes, 137 hypothetical protein-encoding genes, and 439 no-hit genes, while BC01 and MR-4 are similar, with 2,772 known protein-encoding genes, 777 hypothetical protein-encoding genes, and 450 no-hit genes. According to the known coding genes for proteins and enzymes, the genes were involved in three categories, biological processes (37.5%), cellular components (37.5%), and molecular functions (25.0%). The draft genome sequence reported herein will accelerate understanding of the characteristics of this strain and shed more light on the genomic evolution of the *Shewanella* genus.

### Nucleotide sequence accession number.

This whole-genome shotgun project (PRJNA238517) for *Shewanella xiamenensis* BC01 has been deposited in the GenBank under the accession number JGVI00000000.
